# Chronological age and biological maturity are separately positively associated with inhibitory control and working memory in boys and girls

**DOI:** 10.3389/fcogn.2025.1565625

**Published:** 2025-12-12

**Authors:** Luke M. Gilbert, Ryan A. Williams, John G. Morris, Anna Dunn, Ruth Boat, Karah J. Dring, Mary E. Nevill, Simon B. Cooper

**Affiliations:** Department of Sport Science, Sport, Health, and Performance Enhancement (SHAPE) Research Centre, School of Science and Technology, Nottingham Trent University, Nottingham, United Kingdom

**Keywords:** cognitive development, executive function, maturation, adolescence, cognition

## Abstract

Executive function is typically considered from a chronological age perspective, despite the influence of biological maturity on executive function development. The purpose of this study was to investigate the influence of chronological age and biological maturity on inhibitory control and working memory, separately in boys and girls (due to sex differences in biological maturation). The study employed a cross-sectional design and, following familiarization, 736 (400 female) young people (12.3 ± 1.3 years) completed tests of cognitive function on two separate occasions. Participants completed the Stroop test to measure inhibitory control and the Sternberg paradigm to measure working memory. Chronological age and biological maturity were calculated for each participant. Linear regression models were performed separately for boys and girls. Two models were fit for each test and level of executive function: a chronological age model (executive function x chronological age) and a biological maturity model (executive function x biological maturity). Higher chronological age and biological maturity were associated with superior performance on inhibitory control and working memory tests. In boys, the biological maturity models were a significantly better fit (vs. chronological age), whilst in girls, the chronological age models were a better fit (vs. biological maturity). This study provides novel evidence that biological maturity is associated with inhibitory control and working memory. Emphasizing that future investigations into inhibitory control and working memory in young people should consider biological maturity, especially in boys.

## Introduction

Cognitive functions are the brain-mediated functions and processes that allow us to perceive, evaluate, store, manipulate, and make use of information from external and internal sources, before responding to this information (Schmitt et al., 2005). There are six domains of cognitive function: executive function, memory, attention, perception, language, and psychomotor functions (Schmitt et al., 2005).

Executive function refers to a family of top-down mental processes that modulate the operation of various cognitive subprocesses and thereby regulate the dynamics of human cognition (Miyake et al., 2000; Diamond, 2013). These processes enable individuals to plan, organize, and complete everyday tasks (Blakemore and Choudhury, 2006). Importantly, deficits in executive function during childhood have been linked to poorer academic and occupational outcomes (Miller et al., 2012) and to an increased risk of most forms of psychopathology (Snyder et al., 2015). Accordingly, executive function is considered crucial for mental health, physical health, academic success, and overall quality of life (Diamond, 2013). There is general agreement that there are three core executive functions: inhibitory control, working memory, and cognitive flexibility (Miyake et al., 2000; Diamond, 2013). Inhibitory control refers to one's ability to control attention, behavior, thoughts, and/or emotions to override a strong internal predisposition or external lure (Diamond, 2013). Working memory refers to the temporary storage and manipulation of information necessary for complex cognitive tasks (e.g., reasoning, learning, and language comprehension; Baddeley, 1992). Cognitive flexibility helps humans pursue complex tasks and produce adaptable solutions to changing demands (Ionescu, 2012). The executive function abilities are separate but related component processes (Doebel, 2020) involved in flexible, goal-directed behavior (Hackman et al., 2015). Additionally, executive functioning abilities develop throughout childhood, adolescence, and into early adulthood, with each domain following a distinct developmental trajectory that is closely linked to the protracted maturation of the prefrontal cortex, one of the last brain regions to reach full development (Best and Miller, 2010; Arain et al., 2013; Knoll et al., 2016).

Adolescence is a period of substantial biological, social, and cognitive change (Chaku and Hoyt, 2019). The beginning of adolescence occurs around the onset of puberty (Blakemore et al., 2010), although there are sex differences with the onset of puberty occurring, on average, at a younger age in females (11 years; range 8–13 years) than in males (12 years; range 9–14 years; NHS, 2022). The biological changes associated with adolescence include substantial structural and functional development of the brain (Foulkes and Blakemore, 2018). Changes have been observed in gray matter volume, surface area, and cortical thickness, as well as white matter volume and microstructure (Giedd et al., 1999; Vijayakumar et al., 2016; Tamnes et al., 2017). Consequently, cognitive function, specifically the development of executive function, may be hindered or promoted by the timing of puberty onset (Chaku and Hoyt, 2019). Furthermore, structural brain development is not uniform across adolescence, with large variations in both cortical and subcortical regions (Tamnes et al., 2017). The timing of these structural brain developments is further influenced by the sex differences in the onset of puberty, with frontal gray matter reaching its peak at a younger age in females than males (11.0 vs. 12.1 years, respectively; Giedd, 2004). In addition, during adolescence, the prefrontal cortex undergoes synaptic proliferation followed by pruning, a process that supports increased efficiency of executive functioning (Blakemore and Choudhury, 2006; Juraska and Willing, 2017). However, this process may temporarily impair executive function, as pruning disproportionately affects excitatory neurons while sparing inhibitory neurons, creating a transient imbalance (Selemon, 2013). These processes occur earlier in girls than in boys due to earlier pubertal onset, which may contribute to sex differences in the timing of executive functioning development. Therefore, whilst executive function is predominantly investigated with consideration of chronological age, biological maturity could also affect executive function (Laureys et al., 2021).

Whilst it was previously believed that executive functioning abilities were not influenced by biological maturation (i.e., pubertal processes; Luna, 2009), more recent evidence indicates that pubertal processes and associated hormonal changes play an important role (Stumper et al., 2020; França et al., 2025). The sex hormones testosterone, estradiol, and dehydroepiandrosterone drive the physical changes associated with puberty and exert distinct effects on brain development, with differences observed between sexes and brain regions (Laube et al., 2020). For example, Herting et al. (2014) reported that increased testosterone during early puberty was associated with decreased brain volume in boys but increased volume in girls, with more pronounced gray and white matter changes in early vs. later puberty. Similarly, Nguyen et al. (2013) found testosterone predicted decreases in cortical thickness amongst post-pubertal boys, while the association was positive for pre-pubertal girls and reversed after puberty. These findings suggest that the effects of testosterone on brain structure depend on developmental stage and sex, and subsequent work has linked higher levels of pubertal testosterone with lower executive functioning (Nguyen et al., 2017). Taken together, structural and functional brain changes during adolescence are shaped by the timing and level of pubertal hormones, with implications for executive function abilities (Laube et al., 2020). Therefore, examining the influence of biological maturity and executive function is essential for understanding how maturation contributes to cognitive development during adolescence.

To date, the only study to investigate the influence of chronological age and estimated biological maturity on executive function is that of Laureys et al. (2021). Laureys and colleagues (Laureys et al., 2021) concluded that chronological age and biological maturity were positively associated with inhibitory control, working memory, planning, and shifting in adolescent males. For adolescent females, whilst chronological age was positively associated with inhibitory control, working memory, planning, and shifting, biological maturity only influenced inhibitory control. The sex differences in the influence of biological maturity could be due to the females having already passed the onset of puberty and being biologically more mature, and as a result, they may have surpassed the stage of substantial cognitive development associated with the onset of puberty. Resultantly, the influence of biological maturity would be harder to detect in females than in their less biologically mature male counterparts. Consequently, an expanded age range, ensuring a sufficient sample of females close to the onset of puberty, will provide further insight into the influence of biological maturity on executive function. Additionally, in the study by Laureys et al. (2021), the cognitive tasks were performed without control of important confounding variables such as diet and time of day. As a result, the potential influence of these variables on outcomes was not considered.

Therefore, the purpose of this study was to investigate the influence of chronological age and biological maturity across the important executive function sub-domains of inhibitory control and working memory in adolescents. As there are sex differences in biological maturation, the influence of chronological age and biological maturity on executive function will be considered separately in males and females.

## Materials and methods

### Participant characteristics

Data from 745 young people (aged 9–15 years) were collated from previous published (Cooper et al., 2012a,b, 2015, 2016, 2018; Williams et al., 2020; Hatch et al., 2021a,b; Williams et al., 2022a,b; Gilbert et al., 2023a) and unpublished (Cooper, 2012; Dunn, 2023; Gilbert et al., 2023b) studies. These studies either examined inhibitory control alone or a combination of inhibitory control and working memory in young people. Data used in this study were collated from the baseline cognitive function tests in each of these studies (i.e., pre-intervention). All studies were approved by the institutional Human Invasive Ethics Committee. The data for nine participants were removed from the analyses due to insufficient anthropometric data that prevented the calculation of their maturity offset (marker of biological maturity). Therefore, 736 participants were included in subsequent analyses (336 male, 400 female) for inhibitory control, and 560 participants (262 male, 298 female) were included in the working memory analyses, as participants in Cooper et al. (2016) and Dunn (2023) did not complete the Sternberg paradigm (measure of working memory). Descriptive participant characteristics are presented in Table 1.

**Table 1:** Participant characteristics overall and split by sex.

**Variable**	**Overall (*n* = 736)**	**Boys (*n* = 336)**	**Girls (*n* = 400)**
Age (y)	12.3 ± 1.3 [9.0–15.3]	12.1 ± 1.2 [9.4–15.2]	12.4 ± 1.3 [9.0–15.3]
Height (cm)	155.1 ± 10.0 [127.5–191.4]	154.8 ± 10.7 [130.0–191.4]	155.4 ± 9.5 [127.5–183.3]
Body mass (kg)	46.3 ± 11.1 [22.7–95.1]	45.2 ± 11.0 [24.6–83.7]	47.2 ± 11.1 [22.7–95.1]
Body mass index (BMI; kg^.^m^2^)	19.0 ± 3.2 [12.7–35.8]	18.6 ± 3.0 [12.8–33.6]	19.4 ± 3.4 [12.7–35.8]
Maturity offset^a^	−0.36 ± 1.42 [−4.32–3.32]	−1.29 ± 1.05 [−4.32–2.11]	0.42 ± 1.20 [−2.58–3.32]

Data are presented as mean ± SD and [range].

^a^Calculated using the method of Moore et al. (2015).

### Anthropometry

All participants underwent anthropometric measures of body mass, height, and sitting height. Height was measured using a Leicester Height Measure (Seca, Germany), accurate to 0.1 cm, and body mass was measured using a Seca 770 digital scale (Seca, Germany), accurate to 0.1 kg. Sitting height was measured (accurate to 0.1 cm) to allow for the estimation of maturity (by calculating years from peak height velocity) using the method of Moore et al. (2015).

### Chronological age

Chronological age was calculated by subtracting the date of birth from the date of the first experimental trial (Sherar et al., 2007).

### Biological maturity

A common maturity assessment is the determination of years from attainment of peak height velocity (PHV; Sherar et al., 2007), termed maturity offset (Moore et al., 2015). In this study, the sex-specific equations of Moore et al. (2015) were used to calculate years from peak height velocity (maturity offset). The equations of Moore et al. (2015) were used to calculate maturity offset. Therefore, the participant's anthropometric measurements were entered into the relevant sex-specific equation:

Boys


$$\begin{array}{l} \text{Maturity offset }[\text{y}]=-\text{8}.\text{128741 + }(\text{0}.\text{0070346 }\\ \;\times \;(\text{age }\times \text{ sitting height})) \end{array}$$

Girls


$$\begin{array}{l} \text{Maturity offset }[\text{y}]=-\text{7}.\text{709133 + }(\text{0}.\text{0042232}\\ \;\times \;(\text{age }\times \text{ height})) \end{array}$$

A negative value represents the years remaining before attaining PHV, a zero means the time of PHV, and a positive value represents the years that have passed from the attainment of PHV.

### Study design

Following approval from the institution's ethical advisory committee, participants were recruited from primary and secondary schools in the Midlands, UK. Before the commencement of the study, headteacher consent was gained, as per the guidelines for school-based research. Parents/guardians provided written informed consent, and to determine the eligibility of participants for participation, parents/guardians also completed a health screen questionnaire. Written assent was provided by the participants before they participated in the study.

The study employed a cross-sectional design, consisting of familiarization and two main experimental trials that took place at schools. Each main trial was separated by at least 7 days. First, during familiarization, the anthropometric measurements (height, body mass, and sitting height) of the participants were taken. Next, participants were familiarized with the battery of cognitive function tests. Approximately 7 days later, participants completed their first experimental trial. For the first experimental trial, participants attended school following an overnight fast (from 10 pm the previous evening) after consuming a self-selected evening meal. Participants were asked to repeat the same evening meal prior to their second experimental visit. To maintain euhydration, water was permitted *ad libitum* during the fasting period. Participants were instructed to avoid unusually vigorous physical activity for 24 h before each experimental trial. To ensure compliance with these requirements, a member of the research team contacted parents/guardians by telephone or text message the evening before each experimental trial.

On the morning of the first experimental trial, participants reported to the school and consumed a standardized breakfast to control for the potential influence of breakfast on cognitive function in young people (Lundqvist et al., 2019). The standardized breakfast provided 1.5 g of carbohydrate per kg of body mass and consisted of cornflakes, milk, and toast. Thirty minutes post-breakfast, the participants completed the battery of cognitive function tests. Finally, the second experimental trial took place approximately 7 days later and replicated the protocol of the first experimental trial in full.

### Cognitive function tests

The battery of cognitive function tests lasted approximately 8 min and consisted of the Stroop test and Sternberg paradigm, completed in that order on a laptop computer (Lenovo ThinkPad T450; Lenovo, Hong Kong). Instructions for each test and level were presented on the screen before participants began. Before testing, participants were re-familiarized with the tests by completing 3–6 practice stimuli. This procedure helped to reduce potential learning effects. Data for the practice stimuli were discarded and not used in the analyses. The tests were completed in a classroom, with participants seated separately to prevent interaction. The room was kept silent, and participants wore sound-canceling headphones to minimize external disturbance. To enhance screen visibility, the lights in the room were dimmed. Participants were instructed to respond as quickly and accurately as possible.

For each test, the variables of interest were the response time (ms) of correct responses and the proportion of correct responses made. Response times were filtered to exclude unusually slow or fast responses. Minimum (< 100 ms) and maximum (2,000–4,000 ms, depending on task complexity) response times cut-offs were applied. Inverse efficiency scores (IES) were subsequently calculated using mean scores from experimental trials one and two. For each participant, mean response time was divided by the proportion of correct responses (Townsend and Ashby, 1978). IES are a valid method to account for the speed-accuracy trade-off evident in cognitive performance (Vandierendonck, 2017). Separate IES were calculated for each test and level to reflect their differing cognitive demands.

### Stroop test

The Stroop test measures selective attention and executive function, specifically inhibitory control (Miyake et al., 2000). The test has two levels: congruent and incongruent. For both levels, a test word is placed in the center of the screen with a target and a distractor randomly presented on the left and right sides of the screen. Participants selected their responses using the appropriate arrow keys (left or right). On the congruent level, the test, target, and distractor were all presented in a white font. Participants were required to select the word (from the target and distractor) that matched the test word. This level contained 20 stimuli. On the incongruent level (color interference), participants were required to select the color of the word, rather than the word itself (e.g., if “green” was written in blue font, the correct response would be blue). This level contained 40 stimuli. For both levels, choices remained on the screen until the participant responded. The inter-stimulus interval was 1 s.

### Sternberg paradigm

The Sternberg paradigm measures working memory (Sternberg, 1969). It consists of three levels of ascending complexity, requiring participants to retain one, three or five target items. For the one-item level, the number “3” was always the target, with 16 test stimuli presented. For the three and five-item levels, the targets were three (e.g., “A F P”) or five (e.g., “B E H R V”) randomly generated letters. Each level included 32 test stimuli. At the start of each level, target items were displayed along with instructions to press the right arrow key if a target item was presented and the left arrow key otherwise. Correct response was counterbalanced between the left and right arrow keys. For all levels, stimuli were presented in the center of the screen with an inter-stimulus interval of 1 s.

### Statistical analysis

All analyses were performed using RStudio (RStudio Team, 2020). Simple linear regressions were performed using the *glm* function to examine the influence of chronological age and biological maturity on inverse efficiency scores. Due to the sex difference in maturation, simple linear regressions were performed separately for boys and girls. In total, two models were fit for each test and level of executive function and per sex: a chronological age model (executive function x chronological age) and a biological maturity model (executive function x biological maturity). Residual analyses were performed, and if normality or homoscedasticity were violated, the dependent variable was log-transformed, and the residuals were checked thereafter. For models where the dependent variables were log-transformed, the coefficients and 95% CI are presented as % change for a 1-unit increase in the independent variable. The interaction of chronological age and biological maturity on executive function was not examined in multiple linear regression (executive function x chronological age x biological maturity) as the variance inflation factor (VIF) was >10 (as assessed using the *vif* function in R). The chronological age and biological maturity models were compared using the corrected Akaike information criterion (AICc; Akaike, 1998). A lower AICc indicates a better model fit. Akaike weights (AICcWt) were used to compare the probability that each model (chronological age model vs. biological maturity model) was a better fit (Wagenmakers and Farrell, 2004); a higher AICcWt for the biological maturity model indicates a better model fit when compared against the chronological age model. All data are presented as mean ± standard deviation, unless otherwise stated. Statistical significance was accepted as *p* < 0.05.

## Results

Tables 2, 3 present the mean (±SD) inverse efficiency scores (IES) for the Stroop test (inhibitory control) and Sternberg paradigm (working memory) in boys (Table 2) and girls (Table 3), stratified by chronological age groups and biological maturity categories.

**Table 2:** Inverse efficiency scores (IES) for the Stroop test (inhibitory control) and Sternberg paradigm (working memory) for boys, split by chronological age and biological maturity.

**Test**	**Level**	**Overall**	**Chronological age**				**Biological age**					
			**9–10 y**	**11–12 y**	**13–14 y**	>**15 y**	<**-3 y**	−**2.00 to** −**2.99 y**	−**1.00 to** −**1.99 y**	−**0.01 to** −**0.99 y**	**0.00–0.99 y**	>**1 y**
Stroop test (inhibitory control)	Congruent IES	860 ± 165	966 ± 199	864 ± 149	783 ± 124	685 ± 175	946 ± 162	950 ± 187	863 ± 149	821 ± 135	745 ± 124	672 ± 84
	Incongruent IES	1,227 ± 280	1,338 ± 314	1,252 ± 272	1,099 ± 212	958 ± 253	1,350 ± 337	1,349 ± 282	1,242 ± 270	1,176 ± 254	1,019 ± 175	942 ± 160
Sternberg paradigm (working memory)	One-item IES	599 ± 129	644 ± 122	608 ± 136	551 ± 100	542 ± 102	646 ± 148	645 ± 111	602 ± 130	582 ± 129	537 ± 115	493 ± 70
	Three-item IES	764 ± 174	852 ± 207	771 ± 170	701 ± 126	595 ± 70	1,040 ± 822	833 ± 197	792 ± 179	719 ± 137	679 ± 106	569 ± 70
	Five-item IES	973 ± 256	1,130 ± 322	983 ± 243	865 ± 156	692 ± 85	1,103 ± 237	1,135 ± 320	1,004 ± 253	875 ± 153	842 ± 163	713 ± 75

The data presented are mean ± SD.

**Table 3:** Inverse efficiency scores (IES) for the Stroop test (inhibitory control) and Sternberg paradigm (working memory) for girls, split by chronological age and biological maturity.

**Test**	**Level**	**Overall**	**Chronological age**				**Biological age**						
			**9–10 y**	**11–12 y**	**13–14 y**	>**15 y**	−**2.00 to** −**2.99 y**	−**1.00 to** −**1.99 y**	−**0.01 to** −**0.99 y**	**0.00–0.99 y**	**1.00–1.99 y**	**2.00–2.99 y**	>**3 y**
Sroop test (inhibitory control)	Congruent IES	862 ± 181	1,075 ± 163	857 ± 158	792 ± 152	700 ± 70	1,133 ± 171	1,042 ± 168	912 ± 171	842 ± 150	742 ± 110	742 ± 110	655 ± 50
	Incongruent IES	1,189 ± 273	1,455 ± 224	1,195 ± 247	1,077 ± 246	1,104 ± 314	1,530 ± 255	1,408 ± 217	1,272 ± 263	1,173 ± 237	1,080 ± 265	1,045 ± 219	921 ± 214
Sternberg paradigm (working memory)	One-item IES	586 ± 124	716 ± 147	581 ± 107	540 ± 96	508 ± 108	813 ± 165	659 ± 118	633 ± 170	578 ± 101	534 ± 96	502 ± 65	459 ± 67
	Three-item IES	731 ± 146	884 ± 129	734 ± 139	662 ± 108	630 ± 102	970 ± 138	851 ± 125	780 ± 147	746 ± 274	659 ± 103	590 ± 79	572 ± 90
	Five-item IES	898 ± 212	1,132 ± 221	897 ± 200	801 ± 129	748 ± 138	1,174 ± 167	1,120 ± 237	964 ± 210	866 ± 173	802 ± 152	767 ± 153	693 ± 116

The data presented are mean ± SD.

Table 4 summarizes the outcomes of the simple linear regression models, showing the associations of both chronological age and biological maturity with inhibitory control (Stroop test) and working memory (Sternberg paradigm) performance for boys and girls. Coefficients (β) are presented with 95% confidence intervals, alongside measures of model fit (*R*^2^, AICc, and AICc weights) to allow direct comparison between chronological age and biological maturity models.

**Table 4:** Outcome of the simple linear regression models for boys and girls, overall.

**Group**	**Test**	**Level**	**Chronological age model**				**Biological maturity model**				
			β **(95% CI)**	**Sig**	*R* ^2^	**AICc**	β **(95% CI)**	**Sig**	*R* ^2^	**AICc**	**AICcWt** ^b^
Boys	Stroop test	Congruent	−53.93 (−67.15, −40.72)	< 0.001	0.16	4329.86	−66.45 (−81.80, −51.11)	< 0.001	0.18	4323.13	**0.97**
		Incongruent	−73.48 (−96.69, −50.27)	< 0.001	0.10	4708.34	−94.83 (−121.72, −67.94)	< 0.001	0.13	4700.05	**0.98**
	Sternberg paradigm	One item	−25.50 (−37.91, −13.09)	< 0.001	0.06	3277.97	−32.42 (−46.66, −18.19)	< 0.001	0.07	3274.45	**0.85**
		Three item	−46.31 (−62.63, −29.98)	< 0.001	0.11	3421.74	−58.34 (−76.99, −39.70)	< 0.001	0.13	3415.70	**0.95**
		Five item	−78.99 (−102.54, −55.44)	< 0.001	0.14	3613.72	−102.68 (−129.27, −76.09)	< 0.001	0.18	3601.73	**1.00**
Girls	Stroop test	Congruent	−73.27 (−84.99, −61.59)	< 0.001	0.28	5170.99	−78.60 (−91.27, −65.92)	< 0.001	0.27	5172.84	0.28
		Incongruent	−96.39 (−114.81, −77.96)	< 0.001	0.21	5533.24	−103.76 (−123.66, −83.86)	< 0.001	0.21	5533.79	0.43
	Sternberg paradigm	One item^a^	−0.03 (−0.04, −0.03)	< 0.001	0.23	−698.89	−0.03 (−0.04, −0.03)	< 0.001	0.21	−692.04	0.03
		Three item	−61.46 (−72.79, −50.12)	< 0.001	0.24	3726.01	−66.18 (−78.31, −54.06)	< 0.001	0.22	3724.89	**0.64**
		Five item	−90.10 (−106.39, −73.82)	< 0.001	0.28	3941.82	−95.37 (−112.90, −77.83)	< 0.001	0.28	3944.73	0.19

Coefficients (β) are presented in their unstandardized form, with 95% confidence intervals (95% CI).

Coefficient (β) and CI are presented as a 1-unit change in the dependent variable for a 1-unit change in chronological age (1 y) or biological maturity (1 y).

^a^Dependent variable is log-transformed. For log-transformed data, the coefficient and CI are presented as the % change in the dependent variable for a 1-unit change in chronological age (1 y) or biological maturity (1 y).

^b^Probability that the biological maturity model is a better model fit when compared to the chronological age model. Bold denotes a better model fit (i.e., the biological maturity model is a better model fit than chronological age).

### Effect of chronological age and biological maturity on inhibitory control (Stroop test)

In boys, a comparison of the models demonstrated that the biological maturity model (ACCcWt 0.97) was a significantly better fit for congruent Stroop IES [*F*_(1, 334)_ = 72.53, *p* < 0.001]. Biological maturity accounted for 18% of the explained variability in congruent Stroop IES; for each 1 y increase in biological maturity, congruent IES decreased by 66.45 ms (95% CI [51.11, 81.80]).

A comparison of the models for incongruent Stroop IES demonstrated that the biological maturity model (ACCcWt 0.98) was a significantly better fit [*F*_(1, 334)_ = 48.11, *p* < 0.001]. Biological maturity accounted for 13% of the explained variability in incongruent Stroop IES; for each 1 y increase in biological maturity, incongruent IES decreased by 94.83 ms (95% CI [67.94, 121.72]).

In girls, a comparison of the models demonstrated that the chronological age model (ACCcWt 0.72) was a significantly better fit for the congruent Stroop IES [*F*_(1, 398)_ = 151.2, *p* < 0.001]. Chronological age accounted for 28% of the explained variability in congruent Stroop IES; for each 1 y increase in chronological age, congruent IES decreased by 73.27 ms (95% CI [61.56, 84.99]).

A comparison of the models for incongruent Stroop IES demonstrated that the chronological age model (ACCcWt 0.57) was a better fit [*F*_(1, 398)_ = 105.80, *p* < 0.001]. Chronological age accounted for 21% of the explained variability in incongruent Stroop IES; for each 1 y increase in chronological age, incongruent IES decreased 96.39 ms (95% CI [77.96, 114.81]).

### Effect of chronological age and biological maturity on working memory (Sternberg paradigm)

In boys, a comparison of the models demonstrated that the biological maturity model (ACCcWt 0.85) was a significantly better fit for the one-item Sternberg paradigm IES [*F*_(1, 260)_ = 20.10, *p* < 0.001]. Biological maturity accounted for 7% of the explained variability in the one-item Sternberg paradigm IES; for each 1 y increase in biological maturity, one-item IES decreased by 32.42 (95% CI [18.19, 46.66]).

For the three-item level of the Sternberg paradigm, the biological maturity model (ACCcWt 0.95) was a significantly better fit [*F*_(1, 260)_ = 37.98, *p* < 0.001]. Biological maturity accounted for 13% of the explained variability in the three-item Sternberg paradigm IES; for each 1 y increase in biological maturity, three-item IES decreased by 58.34 (95% CI [39.79, 76.99]).

For the five-item level of the Sternberg paradigm, the biological maturity model (ACCcWt 1.00) was a significantly better fit [*F*_(1, 260)_ = 57.83, *p* < 0.001]. Biological maturity accounted for 18% of the explained variability in the five-item Sternberg paradigm IES; for each 1 y increase in biological maturity, five-item IES decreased by 102.68 (95% CI [76.09, 129.27]).

In girls, the chronological age model (ACCcWt 0.97) was a significantly better fit for the one-item Sternberg paradigm IES [*F*_(1, 296)_ = 93.61, *p* < 0.001]. Chronological age accounted for 24% of the explained variability in the one-item Sternberg paradigm IES; for each 1 y increase in chronological age, one-item IES decreased by 3% (95% CI [3, 4%]).

For the three-item level of the Sternberg paradigm, the biological maturity model (ACCcWt 0.64) was a significantly better fit [*F*_(1, 296)_ = 115.40, *p* < 0.001]. Biological maturity accounted for 28% of the explained variability in the three-item Sternberg paradigm IES; for each 1 y increase in biological maturity, three-item IES decreased by 66.18 (95% CI [54.06, 78.31]).

For the five-item level of the Sternberg paradigm, the chronological age model (ACCcWt 0.81) was a significantly better fit [*F*_(1, 296)_ = 118.60, *p* < 0.001]. Chronological age accounted for 28% of the explained variability in the five-item Sternberg paradigm IES; for each 1 y increase in chronological age, five-item IES decreased by 90.10 (95% CI [73.82, 106.39]).

## Discussion

The main findings of the current study were that, in both boys and girls, a higher chronological age and more advanced biological maturity were associated with superior performance on tests of inhibitory control and working memory. However, some key sex differences emerged. In boys, the biological maturity models provided a significantly better fit than chronological age. In girls, the chronological age models appeared to provide a better fit than biological maturity.

A key finding of the present study was that chronological age and biological maturity were both positively associated with inhibitory control and working memory in boys and girls. This is consistent with the findings of Laureys et al. (2021), which to date is the only study to have investigated the influence of chronological age and biological maturity separately on executive function. They reported positive associations of chronological age (boys and girls) and biological maturity (boys) with inhibitory control and working memory. These associations can be explained by the continued development of executive function through childhood, adolescence, and into young adulthood (Huizinga et al., 2006), as evidenced by longitudinal work (Arain et al., 2013). The improvement in inhibitory control and working memory with increasing chronological age into young adulthood is associated with neurological changes (Ferguson et al., 2021), as puberty leads to a second reorganisational period in the prefrontal cortex (Chaku and Hoyt, 2019) and cognitive processing becomes more efficient due to synaptic pruning caused by puberty (Koolschijn et al., 2014). A key difference in the current study is the positive association of biological maturity on both inhibitory control and working memory in girls, whereas Laureys et al. (2021) demonstrated only a positive association of biological maturity with inhibitory control. The disparate findings regarding the influence of biological maturity on working memory in girls could be attributed to the inclusion of female participants of a younger age, who are closer to the onset of puberty, in the current study (9–15 yrs). As the female participants were older (11–16 yrs) in the work of Laureys and colleagues (Laureys et al., 2021), their participants were collectively more mature, potentially making it more difficult to detect the influence of biological maturity on working memory. This is consistent with evidence suggesting that the influence of biological maturation on executive function is most pronounced during early to mid-puberty, after which developmental trajectories tend to converge with chronological age (Gerván et al., 2024).

A further novel finding of the current study is that, following a comparison of models (Table 4), it can be concluded that the biological maturity models were a better fit across all test levels for inhibitory control (Stroop test) and working memory (Sternberg paradigm) in boys. Conversely, in girls, it can be concluded that the chronological age models were a better fit on all test levels for inhibitory control and selective test levels for working memory (the 1-item and 3-item levels on the Sternberg paradigm). These findings are in direct contrast to those of Laureys et al. (2021), who concluded that, when comparing their chronological age and biological maturity models, biological maturity was a better model fit for inhibitory control and working memory in both sexes. The discrepant findings could be attributed to some methodological differences. In the current study, maturity offset was used as an estimation of biological maturity, whereas Laureys et al. (2021) used the Khamis-Roche method (Khamis and Roche, 1994) to determine the percentage of predicted adult height of each participant as their estimation of biological maturation. The Khamis-Roche method is subject to increased error when applied to the age of adolescent growth spurt (11–15 years; Malina et al., 2019). It is recommended that the method be corrected for overestimation when parental height is self-reported (Epstein et al., 1995). These methodological considerations may well have influenced the findings of Laureys et al. (2021). The discrepant findings could also be influenced by the age range of female participants in both studies, as the current study recruited female participants of a broader age range, with more female participants closer to the age of puberty onset. Given that the onset of puberty occurs on average at 11 years in females (range 8–13 years; NHS, 2022), and this onset initiates a period of structural brain reorganization (Peper and Koolschijn, 2012), the participants in Laureys et al. (2021) were likely biologically more mature than those in the current study. Thus, the difference in biological maturity between participant groups could explain the contrasting findings.

**Figure 1:**
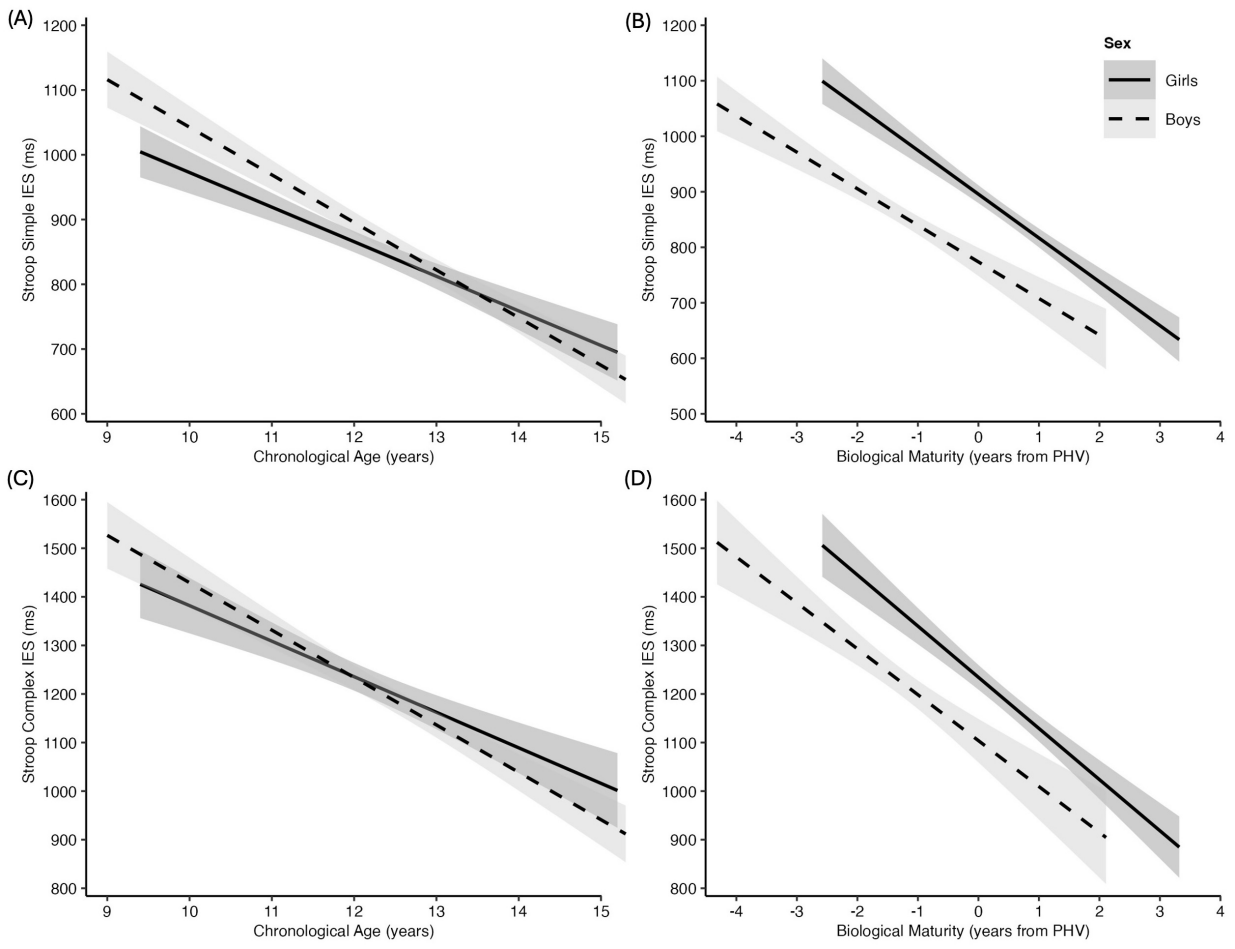
Prediction plots of the means with 95% confidence intervals for the Stroop test. Stroop simple models are presented in plots **(A)** (chronological age model) and **(B)** (biological maturity model); Stroop complex models are presented in plots **(C)** (chronological age model) and **(D)** (biological maturity model). Mean trends are shown with solid black lines for girls and dashed black lines for boys.

**Figure 2:**
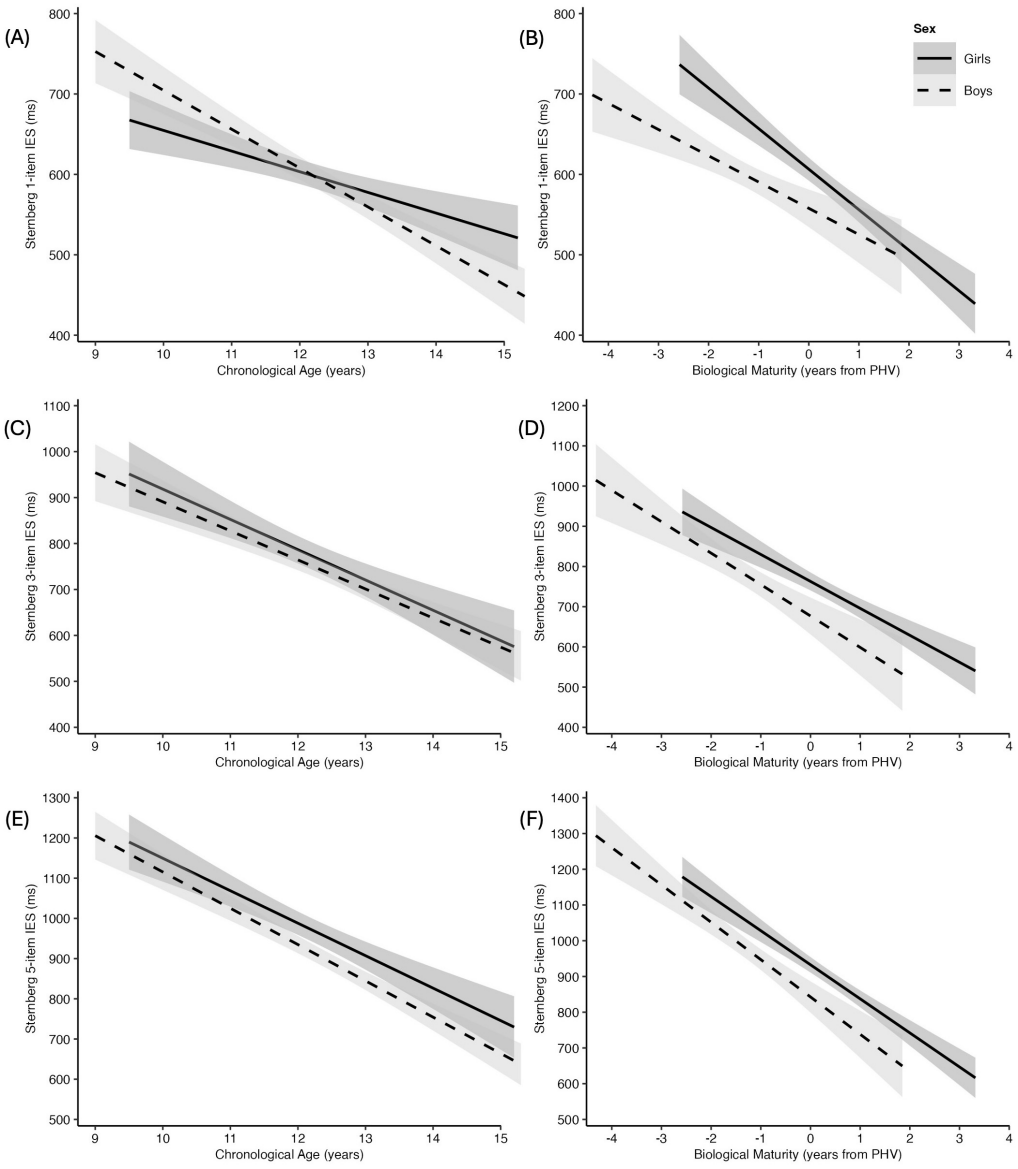
Prediction plots of the means with 95% confidence intervals for the Sternberg paradigm. Sternberg 1-item models are presented in plots **(A)** (chronological age model) and **(B)** (biological maturity model); Sternberg 3-item models are presented in plots **(C)** (chronological age model) and **(D)** (biological maturity model); and Sternberg 5-item models are presented in plots **(E)** (chronological age model) and **(F)** (biological maturity model). Mean trends are shown with solid black lines for girls and dashed black lines for boys.

Within the current study, the comparisons of chronological age and biological maturity models were undertaken separately for each sex. Thus, a comparison of the sex differences in models cannot be undertaken. However, the findings suggest that chronological age was a better model fit for inhibitory control and working memory in girls (except for the three-item Sternberg), whereas biological maturity was a better model fit for inhibitory control and working memory in boys. Additionally, as presented in Figures 1B, D, boys had better IES scores on both levels of the Stroop test at all ages of biological maturity. Furthermore, as presented in Figure 2, boys also had better IES scores for all levels of the Sternberg paradigm at all ages of biological maturity. However, it should be noted that girls had better IES scores on the 3-item and 5-item levels of the Sternberg paradigm at all chronological ages. Girls also performed better by chronological age in the years following the average onset of puberty in girls (11 years) for both levels of the Stroop test and the 1-item level of the Sternberg paradigm (Figures 1, 2). The differences highlighted might be explained by the fact that the onset of adolescence occurs approximately 1–2 years earlier in girls (Koolschijn et al., 2014), initiating a period of structural brain reorganization (Peper and Koolschijn, 2012). Thus, the contrasting model fits between boys and girls could be reflective of the sex differences in the biological maturation process (i.e., on average, boys are of a higher chronological age at any given stage of biological maturity). Future neuroimaging studies should investigate the effect of the brain maturation process on inhibitory control and working memory in both sexes to provide mechanistic insight.

The application of the novel findings from this study should be extended to the emerging body of research investigating inhibitory control and working memory in this population. The emerging body of research includes but is not limited to, exploring the impact of the school climate (Piccolo et al., 2018), natural environments (Vella-Brodick and Gilowska, 2022), adiposity (Williams et al., 2022a), physical fitness (de Bruijn et al., 2018), and both acute (Cooper et al., 2016) and chronic (Dring et al., 2022) physical activity interventions on executive function in young people. Within this body of work, school-based interventions are predominantly undertaken as executive functions are critical for academic achievement (Diamond, 2013), and school-based interventions provide the opportunity to reach nearly all young people (van Slujis et al., 2021). However, school-based interventions are often administered by chronological age, as this is the criterion used to organize education (Van Praag et al., 2018). Thus, these school-based investigations of executive function have not accounted for biological maturity. Yet, there is considerable variation in the biological maturity and the level of biological maturity attained within a school year (Sherar et al., 2007). Adolescent pupils within the same school year will have the same chronological age, yet some will be early maturing, others on time, and others will be late maturing due to the large variability (4–5 years) in puberty onset across individuals (Parent et al., 2003). Therefore, given the influence of biological maturity on inhibitory control and working memory demonstrated in the current study, future school-based investigations examining these domains of executive function in young people should account for biological maturity, especially in boys.

Whilst the present study provides novel insight regarding the sex difference in the influence of chronological age and biological maturity on inhibitory control and working memory, it is not without its limitations. Socioeconomic status, dietary habits, physical fitness, and parental behavior influence executive function during adolescence (Cohen et al., 2016; Stumper et al., 2020; Dandash et al., 2021; Haverkamp et al., 2021), yet these factors were not accounted for in the current analyses. Future studies should therefore examine the potential moderating role of such variables. Additionally, whilst predictions of biological maturity (e.g., Moore's method) offer a non-invasive estimate of maturity status that can be administered on a large scale (i.e., all pupils in a single school year), they have limitations, especially with early and late maturing boys and girls (Malina et al., 2021). The findings of the current study should be interpreted with caution in light of these methodological constraints. A further limitation is that cognitive flexibility was not assessed. As one of the core three executive functions, its exclusion narrows the scope of the findings, as developmental patterns in cognitive flexibility may not mirror those of inhibitory control and working memory. To offer insight beyond the scope of the current study, future work should incorporate neuroimaging evidence of region-specific brain changes and hormonal changes associated with adolescence. Combined with a more robust assessment of biological maturity, this would allow for a more comprehensive understanding of the mechanisms underlying executive function development and how these are shaped by the interplay of biological, environmental, and contextual factors.

## Conclusion

In summary, the present study confirms that, in young people, both chronological age and biological maturity were positively associated with inhibitory control and working memory in boys and girls. However, when the biological maturity model was compared with the chronological age model separately for both sexes, it was clear that the biological maturity model was a better fit for boys and the chronological age model was a better fit for girls. Therefore, future work that investigates inhibitory control and working memory in young people should consider the influence of biological maturity, especially in boys.

## Data Availability

The raw data supporting the conclusions of this article will be made available by the authors, without undue reservation.
